# Maternal common mental disorders and associated factors: a cross-sectional study in an urban slum area of Dhaka, Bangladesh

**DOI:** 10.1186/s13033-017-0129-3

**Published:** 2017-03-14

**Authors:** Ahad Mahmud Khan, Meerjady Sabrina Flora

**Affiliations:** 1 Johns Hopkins University-Projahnmo, Dhaka, Bangladesh; 2 Institute of Epidemiology Disease Control & Research (IEDCR), Dhaka, Bangladesh

**Keywords:** Maternal CMD, Mental health, Factors

## Abstract

**Background:**

Poor maternal mental health has a negative impact on child growth and development. The objective of the study was to find out the associated factors of maternal common mental disorders (CMD) in an urban slum area of Bangladesh.

**Methods:**

This cross-sectional study was carried out from September to November 2013 among conveniently selected 264 mothers having under-five children at Kamrangirchar area of Dhaka. A structured questionnaire based on Self-Reporting Questionnaire-20 (SRQ-20) was used for data collection where a cut-off of 7 was considered to ascertain CMD.

**Results:**

Majority of the mothers were housewives (89.8%), educated up to primary level (40.9%) and lived in nuclear families (83.0%) with low socioeconomic status (64.4%) and moderate household food insecurity (57.5%). The prevalence of maternal CMD was 46.2%. In bivariate analysis, the associated factors of CMD were higher maternal age (*p* = 0.043), lower educational qualification (*p* = 0.015), low socioeconomic status (*p* = 0.004), household food insecurity (*p* < 0.001), maternal undernutrition (*p* = 0.001), child wasting (p = 0.005) and child underweight (p < 0.001). Household food insecurity (*p* < 0.001) and maternal undernutrition (*p* = 0.004) were identified as associated factors of maternal CMD after controlling for socio-demographic variables. There were about 5 times and 12 times increased odds of having maternal CMD in moderately (adjusted OR = 4.8, 95% CI 2.0–11.7) and severely food insecure household (adjusted OR = 11.6, 95% CI 3.5–38.1), respectively, than food secure one. Underweight mothers had 2.5 times increased odds of experiencing CMD as compared with mothers who were not underweight (adjusted OR = 2.6, 95% CI 1.4–5.0).

**Conclusion:**

The prevalence of maternal CMD was relatively higher than other developing countries studied so far. Household food insecurity and maternal under-nutrition were the associated factors of maternal CMD. Therefore, interventions to improve household food security and maternal nutrition can improve maternal CMD and thus make useful contributions to child growth and development.

## Background

Impaired mental health is a neglected public health problem; although more than 450 million people suffer from various forms of mental disorders throughout the world [1]. The overlapping of symptoms of depression, anxiety, fatigue, or somatic complaints lead to grouping of mental disorders under the rubric of common mental disorders (CMD) [2]. It is more prevalent among women especially in the Low and Middle Income Countries (LMICs) [3]. In a study the prevalence of maternal CMD has been reported to range from 21% in Vietnam to 33% in Peru [4]. The prevalence of maternal CMDs is also very high in India (30%) [4] and Bangladesh (49%) [5].

Poor mental health lessens the mother’s capability to take adequate care of her child, which in turn can have adverse effects on child’s growth and development [6, 7]. In a systematic review Surkan et al. has revealed a relationship between maternal depression and child undernutrition [8]. Similar findings are also observed in some other studies [4, 5]. It is possible to lessen child growth retardation up to 30% by reducing prevalence of maternal depression [9].

Mental health is determined not only by biological factors but also socioeconomic and environmental factors [1]. Nguyen et al. has identified some maternal and socioeconomic factors which might play a key role for developing maternal CMD [5]. Harpham et al. has determined some child and household characteristics as the underlying factors of maternal CMD in addition to maternal and socioeconomic factors [4]. In a systematic review Fisher et al. reported CMD is more prevalent among poorer women with gender-based risks or a psychiatric history [3]. Birth of a female child is often a contributing factor for impaired mental health. However education, family support and employment are considered as the protective factors of maternal mental health [10].

Although some studies were conducted related to maternal mental health but this topic has not been previously studied on community based urban population in Bangladesh so far reviewed. Therefore, this study was designed to identify the associated factors of maternal CMDs in an urban area, where interventions should be taken which in turn might improve the child health.

## Methods

### Study design and setting

This cross-sectional study was conducted in an urban slum area at Kamrangirchar of Dhaka district in Bangladesh. Kamrangirchar has a surface area of 3.68 km^2^ with an estimated population of 400,000.

### Study population, sample size and sampling strategy

The study population consisted of mothers with children aged 0–5 years. Pregnant mothers were excluded from the study. The sample size was 264. It was calculated taking into consideration the prevalence of maternal CMD as 49% [5] with allowable error of 6% and confidence interval of 95%. Total data collection period was from September to November 2013. Households were selected by convenience sampling. The convenience sampling was used because of poorly demarcated and often unnamed lanes characteristic in the densely populated slum; therefore community mapping and probability sampling were not feasible. Participants were selected in the community by knocking at the door and collecting the information about the availability of under five children and their mothers. If more than two under-five children were present youngest one was selected.

### Research instruments

A set of structured questionnaire in Bangla was used for data collection. It included questions on socio-demographic variables, questions from Self-Reporting Questionnaire-20 (SRQ-20) [11], Household Food Insecurity Access Scale (HFIAS) [12], and Kuppuswamy’s socioeconomic status scale [13]. Checklist for height and weight was used.

The SRQ-20, a screening tool, developed by World Health Organization (WHO) was used to assess mental health of the mothers. It consists of twenty yes/no items asking about the experience of anxiety, depressive, panic and somatic symptoms within last 30 days. The score of each item is either 1 or 0. A score of 1 specifies that the symptom was present during past 30 days; a score of 0 specifies that the symptom was not present. The sum of the twenty scores generates an overall SRQ-20 scale, where higher scores direct poor mental health and vice versa [11]. It is a valid, reliable and adaptable for screening mental disorders in the developing countries [4–6, 14, 15]. A cut-off of 7 was considered to ascertain maternal CMD, as recommended by some other studies [4, 5].

HFIAS which is appropriate for urban population was used to measure household food security. Questions on behavior and perceptions related to household food insecurity are asked for a reference period of 4 weeks preceding the interview. Food secure, mildly food insecure, moderately food insecure and severely food insecure households are categorized by this tool [12].

Modified Kuppuswamy’s socioeconomic status scale was used to assess socioeconomic status that included three variables: Family head’s education, occupation and monthly family income. Based on total score socioeconomic status was categorized as upper (total score 26–29), upper middle (total score 16–25), lower middle (total score 11–15), upper lower (total score 5–10) and lower (total score <5) classes [13]. Monthly family income was estimated in Bangladeshi Taka (BDT) where 1 US dollar was equivalent to 79 BDT.

Child nutritional status was estimated using WHO recommended height for age z-score (HAZ), weight for height z-score (WHZ) and weight for age z-score (WAZ) [16] while maternal undernutriton was defined as body mass index (BMI) <18.5 kg/m^2^ [17]. Child illness was measured through maternal recall of symptoms of diarrhea and acute respiratory infection (ARI) in the 14 days prior to the interview.

### Data collection

Face-to-face interview and measurement of the height and weight of child and mother were done for data collection. Interview was taken at home of the participant ensuring the privacy and confidentiality as far as possible. Before the interview, the detail of the study was explained to each eligible respondent and written informed consent was taken. Standard procedure was followed for the measurement of child’s length/height and weight. Supine length was measured up to age of 24 months and standing height was measured after 24 months. Mother’s height and weight were also taken.

### Data processing and analysis

After completion of data collection, each question was checked for completeness. Statistical software SPSS version 21 was used for data analysis. It started with descriptive analysis. Means and standard deviations were calculated for continuous variables while frequencies and percentages were calculated for categorical variables, simultaneously bivariate analysis was done to find out the association. *χ* ^2^ test, Fisher’s Exact test and t test were carried out to assess the association of maternal CMD with socio-demographic, household characteristics and child characteristics which one was appropriate. When significant association was found, odds ratio (OR) and corresponding 95% confidence interval (CI) were calculated to determine the strength of association. Forward Logistic Regression (LR) was done to find out the factors associated with maternal CMD. Forward LR starts with no variables in the model, testing the addition of each variable using a chosen model fit criterion, adding the variable whose inclusion gives the most statistically significant improvement of the fit, and repeating this process until none improves the model to a statistically significant extent. Statistical significance was defined as *p* < 0.05.

## Results

### Baseline characteristics of the respondents

The age of the mothers ranged from 15 to 44 years with a mean of 25.30 ± 5.65 years. Maximum mothers (40.9%) were educated up to primary level and more than two-third of the fathers were illiterate or educated up to primary level (73.1%). Most of the mothers (89.8%) were housewives, the rest were working mothers. More than one-fourth of the fathers (28.4%) lied in the group of unskilled workers and same proportion (28.4%) was in the group of service and small business.

Majority (64.4%) of the respondents were found in low socioeconomic status. More than half (52.7%) of the respondents were fallen at the category of moderately food insecure. About one-tenth (12.9%) of the respondents were at food secure state. The monthly family income of the respondents ranged from 2000 BDT to 50,000 BDT. Average income was 10,595 ± 5234 BDT. Most of the respondents (83%) belonged to nuclear family. Total family member ranged from 3 to 12 and average family size was 4.5.

Infants were relatively less common (14.0%) than other age groups whereas the age group 24–35 months consisted of maximum number of children (31.4%). Sex distribution of the children was almost equal with a ratio of 1:1.2.

### Factors associated with maternal CMD

Out of 264 mothers, 122 (46.2%) were found having CMD. Among the maternal factors, age (*p* = 0.043), educational status (*p* = 0.015) and nutrition (*p* = 0.001) were significant associated with CMD. Mothers aged over 35 years had three times increased odds of having CMD than in those of other age groups (OR = 3.27, 95% CI 1.10–9.75). Proportion of it decreased with the educational attainment of the mothers. It was about 2 and 4 times decreased odds in the mothers with class 6–8 (OR = 0.45, 95% CI 0.22–0.93) and ≥class 9 (OR = 0.25, 95% CI 0.10–0.67) of education, respectively, than the illiterate mothers. CMD was almost equal between housewives (46.0%) and working mothers (48.1%). Underweight mothers had about 2.5 times increased odds of having CMD than the normal mothers (OR = 2.66, 95% CI 1.44–4.90) (Table 1).

**Table 1:** Results of bivariate analyses of association between maternal characteristics and maternal CMD

Characteristics	Maternal CMD		*p* value^†^	OR (95% CI)
	No	Yes		
	N (%)	N (%)		
Age of the mother in years				
<20^a^	17 (56.7)	13 (43.3)	0.043	
20–24	54 (53.5)	47 (46.5)		1.14 (0.50–2.59)
25–29	42 (63.6)	24 (36.4)		0.75 (0.31–1.80)
30–34	21 (53.8)	18 (46.2)		1.12 (0.43–2.92)
≥35	8 (28.6)	20 (71.4)		3.27 (1.10–9.75)
Educational status of mother				
Illiterate^a^	31 (43.1)	41 (56.9)	0.015	
Primary	55 (50.9)	53 (49.1)		0.73 (0.40–1.33)
Class 6–8	35 (62.5)	21 (37.5)		0.45 (0.22–0.93)
≥Class 9	21 (75.0)	7 (25.0)		0.25 (0.10–0.67)
Occupation of mother				
Housewife^a^	128 (54.0)	109 (46.0)	0.831	
Working	14 (51.9)	13 (48.1)		1.09 (0.49–2.42)
Nutrition of mother				
Normal^a^	122 (58.9)	85 (41.1)	0.001	
Underweight	20 (35.1)	37 (64.9)		2.66 (1.44–4.90)

^†^χ^2^ test

^a^Reference group

Among the household characteristics, socioeconomic status (*p* = 0.004), household food security (*p* < 0.001) and family income (*p* = 0.001) were associated with maternal CMD. Proportion of maternal CMD decreased with improved socioeconomic status. It was about 6 times decreased odds in mothers of upper middle class than those of lower and lower middle class (OR = 0.17, 95% CI 0.05–0.61). Percentage of poor mental health increased with increased food insecurity access. Maternal CMD was found about 5 times and 12 times more in moderately (OR = 4.79, 95% CI 1.96–11.74) and severely (OR = 11.57, 95% CI 3.51–38.14) food insecure access households, respectively, than those with food secure one. On average, monthly family income was less in the mothers of poor mental health (BDT 9492 ± 4049) than normal one (BDT 11,542 ± 5923). Family characteristics i.e. type of family, size of family and frequency of under-five children were not associated with maternal CMD (Table 2).

**Table 2:** Results of bivariate analyses of association between household characteristics and maternal CMD

Characteristics	Maternal CMD		*p* value^†^	OR (95% CI)
	No	Yes		
	N (%)	N (%)		
Socioeconomic status				
Lower^a^	81 (47.5)	89 (52.5)	0.004	
Lower middle	45 (60.0)	30 (40.0)		0.61 (0.35–1.05)
Upper middle	16 (84.2)	3 (15.8)		0.17 (0.05–0.61)
Household food security				
Food secure^a^	27 (79.4)	7 (20.6)	<0.001	
Mildly food insecure	46 (73.0)	17 (27.0)		1.43 (0.52–3.88)
Moderately food insecure	62 (44.6)	77 (55.4)		4.79 (1.96–11.74)
Severely food insecure	7 (25.0)	21 (75.0)		11.57 (3.51–38.14)
Monthly family income in BDT				
Mean ± SD	11,542 ± 5923	9492 ± 4049	0.001ˆ	
Family type				
Nuclear family^a^	113 (51.6)	106 (48.4)	0.115	
Joint family	29 (64.4)	16 (35.6)		0.59 (0.30–1.14)
Family size				
Small^a^	87 (55.4)	70 (44.6)	0.753	
Medium	41 (52.6)	37 (47.4)		1.12 (0.65–1.93)
Large	14 (48.3)	15 (51.7)		1.33 (0.60–2.93)
No. of under five children				
One child^a^	129 (56.1)	101 (43.9)	0.051	
More than one	13 (38.2)	21 (61.8)		2.06 (0.985–4.32)

^†^χ^2^ test, ˆt test

^a^Reference group

Age, sex and illness of the child were not associated with maternal CMD. However, child nutrition was significantly associated with maternal CMD. Mother of the wasting child had about 2.5 times increased odds of suffering from CMD than the mother of normal child (OR = 2.51, 95% CI 1.31–4.80). Again, mother of the underweight child had about 2.6 times increased odds of suffering from CMD than the mother of normal child (OR = 2.61, 95% CI 1.54–4.41) (Table 3).

**Table 3:** Results of univariate analyses of association between child characteristics and maternal CMD

Characteristics	Maternal CMD		*p* value^†^	OR (95% CI)
	No	Yes		
	N (%)	N (%)		
Sex of the child				
Male^a^	69 (56.6)	53 (43.4)	0.403	
Female	73 (51.4)	69 (48.6)		1.23 (0.76–2.00)
Age of the child in months				
<6^a^	17 (68.0)	8 (32.0)	0.526	
6–11	5 (41.7)	7 (58.3)		2.98 (0.72–12.34)
12–23	37 (54.4)	31 (45.6)		1.78 (0.68–4.68)
24–35	45 (54.2)	38 (45.8)		1.79 (0.70–4.62)
≥36	38 (50.0)	38 (50.0)		2.13 (0.82–5.51)
Child illness				
Diarrhoea				
No^a^	35 (49.3)	36 (50.7)	0.068	
Yes	22 (33.8)	43 (66.2)		1.90 (0.95–3.80)
ARI				
No^a^	52 (42.6)	70 (57.4)	0.410	
Yes	4 (30.8)	9 (69.2)		1.67 (0.49–5.73)
Child nutrition				
HAZ				
Normal^a^	86 (58.5)	61 (41.5)	0.086	
Stunting	56 (47.9)	122 (46.2)		1.54 (0.94–2.51)
WHZ				
Normal^a^	125 (57.9)	91 (42.1)	0.005	
Wasting	17 (35.4)	31 (64.6)		2.51 (1.31–4.80)
WAZ				
Normal^a^	108 (61.7)	67 (38.3)	<0.001	
Underweight	34 (38.2)	55 (61.8)		2.61 (1.54–4.41)

^†^χ^2^ test

^a^Reference group

Forward LR was performed to find out the factors associated with maternal CMD. The final model contained seven independent variables (maternal age, education, nutrition, household food security, monthly family income, child WHZ and WAZ). The full model containing all predictors was statistically significant (*p* < 0.001). As shown in Table 4 only two of the independent variables made a unique statistically significant contribution to the model (household food security and maternal nutritional status). The best predictor of maternal CMD was household food security. Maternal CMD was about 5 times and 12 times more likely in moderately (adjusted OR = 4.79, 95% CI 1.96–11.74) and severely food insecure household (adjusted OR = 11.57, 95% CI 3.51–38.14), respectively, than food secure one after controlling for all other factors in the model. The odds ratio for maternal nutrition indicated that underweight mothers were about 2.5 times more likely to suffer from poor mental health than normal one (OR = 2.61, 95% CI 1.36–5.04).

**Table 4:** Multivariate analysis to find out the best predictors of maternal mental health

Characteristics	*p* value	Adjusted OR^b^ (95% CI)
Household food security		
Food secure^a^	<0.001	
Mildly food insecure		1.43 (0.52–3.88)
Moderately food insecure		4.79 (1.96–11.74)
Severely food insecure		11.57 (3.51–38.14)
Nutrition of the mother		
Normal^a^	0.004	
Underweight		2.61 (1.36–5.04)

^a^Reference group

^b^Adjusted for maternal age, education, socioeconomic status, monthly family income and child WHZ and WAZ

## Discussion

Out of 264 mothers, 122 (46.2%) were found suffering from CMD. This result is almost similar (49%) to a previous study conducted in Bangladesh [5]. Although CMD is very common in developing countries, its prevalence varies from country to country—Vietnam 31.2%, Ethiopia 39.4% [5], Peru 30% and India 30% [4]. Mental health of the mother was evaluated by WHO recommended SRQ-20. The reliability and validity of this instrument are well established [11] and it has been used in several studies [4, 5, 14, 15].

Similar to the study of Nguyen et al. and Harpham et al., this study found significant association between higher maternal age and CMD [4, 5]. It indicates that older age might have an effect on the mental health. Current study findings showed that educational level plays a significant role on maternal mental health which is consistent with the study of Nguyen et al. [5]. Underweight mothers were more prone to suffer from CMD, reported by this study. Association of maternal nutrition and mental health persisted even after controlling for other factors in multivariate analysis. This finding is consistent with a previous study finding [5]. It indicates that intervention on maternal nutrition should be considered to improve mental health of mothers.

As the study was conducted in an urban setting, modified Kuppuswamy’s socioeconomic scale was considered which was appropriate for urban population only. Majority (64.4%) of the respondents were found in low socioeconomic class. Association was found between socioeconomic status and maternal mental health in univariate analysis, though it disappeared in multivariate analysis. Maternal CMD was more common in lower socioeconomic class. It is similar with another study finding [5]. Harpham et al. finds the association of wealth index with maternal mental health [4].

HFIAS was used in this study to measure food security status [12]. HFIAS is applicable for both rural and urban population and it has been used in national surveys, and the reliability and validity are well established [5, 12]. About 13% of the households were at food secure state and others were in various level of food insecurity. This result is lower than the result found in another study in Bangladesh [5] because the study population represented low socioeconomic status. Household food security was identified as best predictor of maternal mental health in this study after controlling for the effects of other variables in multivariate analysis. Mothers were 5 times and 12 times more likely to suffer from CMD in moderately and severely food insecure families, respectively, than in food secure families. Similar finding is reported by Nguyen et al. [5] which indicates that emphasis on food security is necessary as an intervention to improve maternal mental health.

No relationship was reported between maternal mental health and child illness in this study. To our knowledge, only few studies examined the association between maternal mental health and child illness [5, 18, 19]. Nguyen et al. discovers association of maternal mental health with both diarrhea and ARI [5]. The reason might be data were collected in a short period of time and here season might play a role. This study provided evidence of an association of child wasting and underweight with maternal mental health. It thus confirmed previous evidence from some other countries [4, 5, 14, 15].

Despite all effort there were some limitations in this study. The relationship observed in this study was based on a cross-sectional study. Therefore, the association found does not reflect causal association. Although important socioeconomic characteristics were adjusted, it is difficult to capture all relevant aspects; thus residual confounding is still less likely. Data were collected based on recall of the mothers’ mental distress in the 1 month before to the interview. There might be either under or over-reporting of symptoms as recall has few limitations for data collection. SRQ-20 is a screening tool to assess mental health, not a diagnostic one. Therefore, the number of mothers, identified as CMD, might differ from the actual number. Another limitation was convenience sampling technique was applied to recruit the respondents, which limits the generalizability of the results to other population.

## Conclusion

Maternal CMD was relatively higher than other developing countries especially among the urban population. Household food security and maternal nutrition were the best predictors of maternal mental health. The study result may contribute to understand the importance of maternal mental health as a public health problem that may help to prevent, detect and treat maternal mental health aimed at improving child health and nutrition.

## Abbreviations

ARI: acute respiratory infection
BDT: Bangladeshi Taka
BMI: body mass index
CMD: common mental disorders
HAZ: height for age z-score
HFIAS: Household Food Insecurity Access Scale
LMIC: Low and Middle Income Countries
LR: logistic regression
NIPSOM: National Institute of Preventive and Social Medicine
SPSS: Statistical Package for Social Sciences
SRQ: Self-Reporting Questionnaire
WAZ: weight for age z-score
WHO: World Health Organization
WHZ: weight for height z-score
